# Supplementary material for: PD-L1 signaling in reactive astrocytes counteracts neuroinflammation and ameliorates neuronal damage after traumatic brain injury
Source: J Neuroinflammation. 2022 Feb 8;19:43. doi: 10.1186/s12974-022-02398-x (PMC8822654; doi:10.1186/s12974-022-02398-x)
Supplement: Supplementary file 1 — Additional file 1: Figure S1. PD-L1 Ab via subcutaneous injection reached the injured site and bound to GFAP+ reactive astrocytes in the brain of TBI mice. Mice 24 h post-TBI were given PD-L1-Alex647-conjugated Ab (red) or IgG via SC injection. Brain tissues were harvested 48 h post-injection for IHC analysis of PD-L1 binding. Left panels, the high power images showing that there is no red signal (PD-L1) detected in the brain of TBI mice brain with IgG treatment. Right panels, the high power images showing that PD-L1-Alex647-conjugated Ab (red) reached the injured site and bound to GFAP+ reactive astrocytes. Similar results were obtained from 3 mice of each group. Figure S2. No significant change of GFAP expression with PD-L1 Ab treatment post-TBI. One week post-TBI (6 days after PD-L1 Ab or IgG treatment), GFAP expression in injured cortex was examined using IF staining. A) and B), GFAP-positive reactive astrocytes (green signal) in injured cortex at 1 week post-TBI with IgG control or PD-L1 Ab treatment. There was no significant difference of GFAP signal between groups. A1) and B1), High power images from the white box of A) and B) showed that PD-L1 Ab treatment had no effect on the morphology of GFAP-positive reactive astrocytes. C), GFAP intensity quantification confirmed that PD-L1 Ab treatment did not change the GFAP expression after TBI. ns, not significant. [file 12974_2022_2398_MOESM1_ESM.docx]

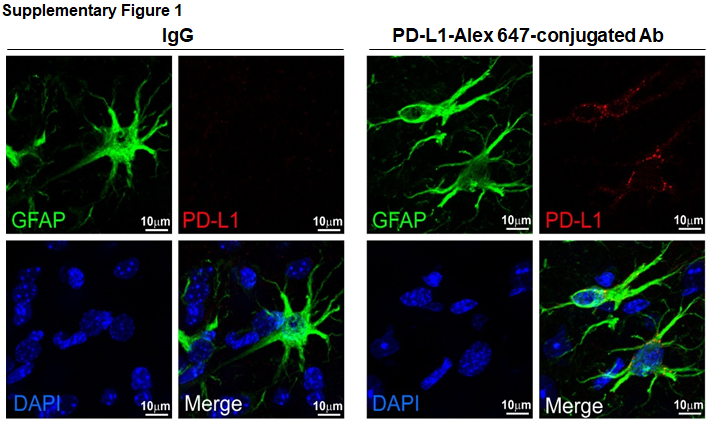


**Figure S1. PD-L1 Ab via subcutaneous injection reached the injured site and bound to GFAP^+^ reactive astrocytes in the brain of TBI mice.** Mice 24 h post-TBI were given PD-L1-Alex647-conjugated Ab (red) or IgG via SC injection. Brain tissues were harvested 48 h post-injection for IHC analysis of PD-L1 binding. Left panels, the high power images showing that there is no red signal (PD-L1) detected in the brain of TBI mice brain with IgG treatment. Right panels, the high power images showing that PD-L1-Alex647-conjugated Ab (red) reached the injured site and bound to GFAP^+^ reactive astrocytes. Similar results were obtained from 3 mice of each group.

**Figure S2**


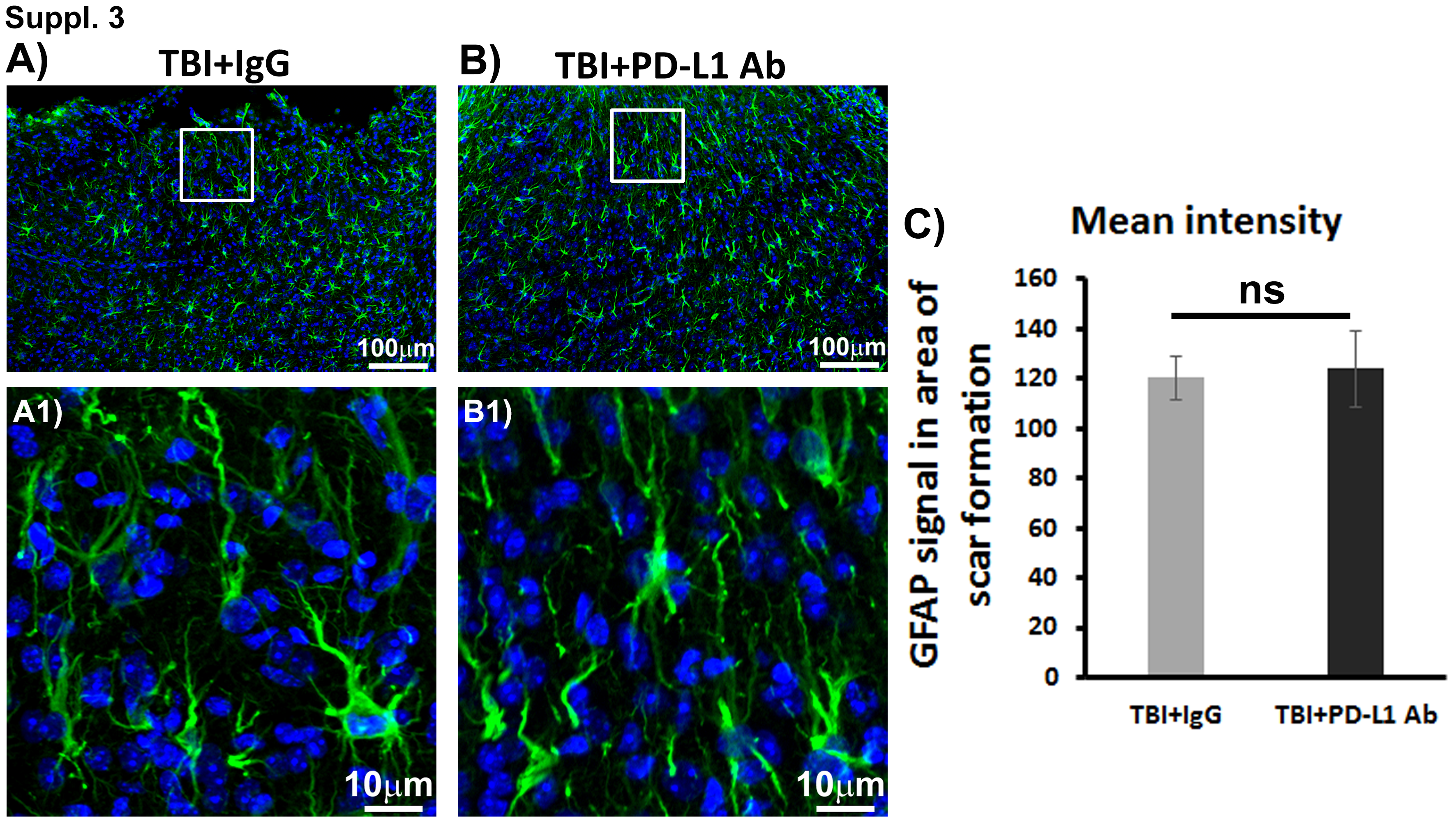


**Figure S2:** No significant change of GFAP expression with PD-L1 Ab treatment post-TBI. One week post-TBI (6 days after PD-L1 Ab or IgG treatment), GFAP expression in injured cortex was examined using IF staining. **A)** and **B)**, GFAP-positive reactive astrocytes (green signal) in injured cortex at 1 week post-TBI with IgG control or PD-L1 Ab treatment. There was no significant difference of GFAP signal between groups. **A1)** and **B1)**, High power images from the white box of **A)** and **B)** showed that PD-L1 Ab treatment had no effect on the morphology of GFAP-positive reactive astrocytes. **C)**, GFAP intensity quantification confirmed that PD-L1 Ab treatment did not change the GFAP expression after TBI. ns, not significant.
